# Ichnological evidence of Megalosaurid Dinosaurs Crossing Middle Jurassic Tidal Flats

**DOI:** 10.1038/srep31494

**Published:** 2016-08-19

**Authors:** Novella L. Razzolini, Oriol Oms, Diego Castanera, Bernat Vila, Vanda Faria dos Santos, Àngel Galobart

**Affiliations:** 1 Mesozoic Research Group, Institut Català de Paleontologia ‘Miquel Crusafont’, C/ Escola Industrial 23, 08201 Sabadell, Catalonia, Spain; 2 Universitat Autonoma de Barcelona, Facultat de Ciències (Geologia), 08193, Bellaterra (Spain); 3 Bayerische Staatssammlung für Paläontologie und Geologie and GeoBioCenter, Ludwig-Maximilians-Universität, Richard-Wagner-Str. 10, 80333 Munich, Germany; 4 Museu de la Conca Dellà, carrer del Museu, 4, 25650, Catalonia, Spain; 5 Museu Nacional de História Natural e da Ciência – Universidade de Lisboa, Rua da Escola Politécnica, 58, 1250−102 Lisboa, Portugal

## Abstract

A new dinosaur tracksite in the Vale de Meios quarry (Serra de Aire Formation, Bathonian, Portugal)preserves more than 700 theropod tracks. They are organized in at least 80 unidirectional trackways arranged in a bimodal orientation pattern (W/NW and E/SE). Quantitative and qualitative comparisons reveal that the large tridactyl, elongated and asymmetric tracks resemble the typical Late Jurassic-Early Cretaceous *Megalosauripus* ichnogenus in all morphometric parameters. Few of the numerous tracks are preserved as elite tracks while the rest are preserved as different gradients of modified true tracks according to water content, erosive factors, radial fractures and internal overtrack formations. Taphonomical determinations are consistent with paleoenvironmental observations that indicate an inter-tidal flat located at the margin of a coastal barrier. The *Megalosauripus* tracks represent the oldest occurrence of this ichnotaxon and are attributed to large megalosaurid dinosaurs. Their occurrence in Vale de Meios tidal flat represents the unique paleoethological evidence of megalosaurids moving towards the lagoon, most likley during the low tide periods with feeding purposes.

Megalosaurid dinosaurs were the dominant tetanuran theropods in the Middle Jurassic age^1^, a time period generally featured by the scarcity of dinosaur fossils worldwide^2^. For this period of time, most of the theropod European record is assigned to the Megalosauridae family based on skeletal remains from France, England and Scotland^3^,^4^,^5^,^6^,^7^,^8^,^9^. In addition, the ichnological record, mostly concentrated in England, Scotland and Portugal^10^,^11^,^12^,^13^ preserves various large track morphotypes that fit into the approximate size of *Megalosaurus*, a characteristic mid-to-large basal megalosaurid from the Bathonian of England^6^,^7^. The Lusitanian basin in West-Central Portugal bears two temporally significant theropod tracksites of Bathonian age: Algar dos Potes and Vale de Meios tracksites. Because of the poor dinosaur record in the Middle Jurassic, the description of new localities represents a very significant contribution to understand the composition of dinosaur faunas of that age. Particularly, the occurrence of new fossil evidence potentially related to megalosaurid theropods increases the knowledge of the clade in terms of diversity, taxonomy, behaviour and environmental distribution. New data from tracks and trackways is also of pivotal importance to ascertain trackmaker affinity and habitat. The aim of the present study is to formally describe the Vale de Meios tracksite (Figs 1, 2, 3), one of the largest theropod tracksites described worldwide from the Middle Jurassic. For this purpose we provide a detailed sedimentary analysis and an exhaustive description of the track morphology, preservation and ichnotaxonomy. Moreover, paleoenvironmental and paleoethological reconstructions are provided on the basis of the unique orientation and arrangement of the trackways on the tidal flat.[Fig f2]

## Materials and Methods

Two field campaigns in 2014 and 2015 produced a 2D cartography and photogrammetric models of the most interesting trackways and track morphologies (see Supplementary Information for three-dimensional models) at the Vale de Meios locality (Fig. 3). The whole outcrop was divided into 5 × 5 m squares and each square was provided with a letter and a number in order to locate tracks with x, y coordinates (Fig. 3A). Photogrammetric models of tracks and three trackways (VM1, VM2 and VM3; Fig. 3B) were undertaken with Canon PowerShot G12 camara (focal length 6 mm, 3648 × 2432 resolution) following the general methodology of Mallison and Wings^14^. Point clouds were processed in AgisoftPhotoscan standard version 1.1.4. build 2021 software (http://www.agisoft.ru/). Photogrammetric models presented in this work count on 14 photos for track VMX.1 (0.6 mm of resolution), 15 photos for track VMX.2 (0.6 mm resolution), 209 photos for trackways VM1 and VM2 (2.25 mm of resolution) and 229 photos for trackway VM3 (2.25 mm of resolution). All these models are available as Supplementary Information files. Three-dimensional models were converted to colour maps in the open source CloudCompare software (v.2.6.1, http://www.danielgm.net/cc/). Contour lines (isolines) were obtained in free software Paraview 4.4.0 version (http://www. paraview.org/), importing scaled and oriented models with respect to the Z axis from CloudCompare (v.2.6.1) and they were set every 0.8 mm distance according to maximum and minimum heights of the plane where tracks are. Track length (TL) and width (TW), track ratio (TL/TW), interdigital angles II^III and III^IV, pace length (PL), stride length (SL), pace angulation (ANG) were measured from trackway photogrammetries (Tables 1 and 2). Furthermore, in order to compare individual tracks, we calculated the anterior triangle ratio^15^ as a way to explore the morphodynamic relationship between the mesaxonic index and the anterior shape of the studied tracks. The anterior triangle (AT) is an index measured from the distal point on the digital pads of digits II, III and IV and not from claw marks, which may be variably preserved^15^. The maximum height of the triangle is measured perpendicular to the transverse base of the triangle and expressed as the l/w ratio (AT l/w).

Sediment samples (IPS87258, IPS87264, IPS87259) were collected both on the track surface level and the infill inside the tracks (squares A10 and B5, Fig. 3A), and 10 thin sections were prepared for sedimentological (microfacies) and environmental determinations.

### Geological and Geographical setting

The Vale de Meios locality is found in the Middle Jurassic micritic limestones from the Maciço Calcário Estremenho (Limestone Massif of Estremadura, Lusitanian Basin), which encompasses the relief area of the central-west part of Portugal. The strata containing the analysed track were deposited in the eastern margin of the Protoatlantic Ocean, formed as a result of the rifting that started in the Midde Jurassic. At those times, the western part of the Iberian plate (present Portugal) contained the Lusitanian Basin, infilled by shallow marine carbonates (limestones and dolostones) and in the lower part by marly-limestones and marls^16^,^17^.

Sedimentologically, the Middle Jurassic series from Portugal mainly include high-energy deposits originated in barrier-islands paleoenvironments and lagoonal and peritidal deposits formed within the protected areas of the internal back-barrier. Azerêdo *et al*.^18^ suggested a depositional model for the Middle Jurassic of the Lusitanian Basin with an E/SE to W/NW carbonated-ramp system. During the Bajocian-Bathonian interval (Fig. 2), the eastern part of the basin was characterized by margino-marine and confined lagoon environments suggesting a system progradation from east to west^16^. The barrier island environment is represented by the Santo António-Candeeiros Formation, while the associated lagoonal and peritidal ones are represented by the Serra de Aire Formation. This last formation contains the Vale de Meios tracksite here reported, which is Bathonian in age after the occurrence of agglutinated foraminifera (i.e. *Alzonella cuvillieri*^19^).

Our sedimentological observations are in agreement with this scheme^16^. In the tracking surface two different kinds of limestones are present: a) massive limestone where footprints are produced; b) laminated limestone found as internal overtracks (*sensu* Marty^20^). Each of these two different types has distinct features when observed in thin sections (see examples in Fig. 4).

- *Massive limestone* (IPS87258, Fig. 4A; IPS87264, Fig. 4B). They correspond to grainstones^21^ with pellets, ostracods tests and agglutinated foraminifera as main components. Although both fossils are found together, there is always a dominant one. When ostracodes are abundant (Fig. 4A), foraminifera are scarse and viceversa (Fig. 4B). This suggests small salinity variations within a similar environment, since ostracods are rather euryhaline (i.e, tolerant to such variations) if compared with foraminifera, which are more stenohaline (less tolerant), see pag. 618 of Flügel^22^.No lamination is observed. Sparitic and micritic matrix are found, therefore this microfacies can be classified both as pelbiosparite and pelbiomicrite, respectively^23^. In fact, both kinds of matrix are observed in a single thin section (Fig. 4A,B).
- *Laminated limestone* (IPS87259, Fig. 4C,D). They correspond to mudstones^21^ where ostracodes tests are abundant and foraminifera are absent. Micrite aggregates (peloids) are found and no strict pellets are observed. According to the components, this microfacies can be classified as pelbiomicrites^23^. Lamination is also visible in thin section (Fig. 4C) as clotted micrite layers with irregularly elongated, laminated fenestral pores (probably resulting from the deterioration of organic matter). This microfacies contains small isolated unimodal and euhedral relics of rhombohedrons, which are likley to have belonged to dolomite crystals^24^ (see Fig. 4D).

Both microfacies would belong to the standard microfacies SMF 16: a) non-laminated peloid grainstone and packstone and b) laminated peloidal bindstone^22^.

As a general observation, both microfacies display no mud-cracking evidence, meaning that the tracking surface did not undergo a strong dessication and therefore the tracking surface was a moisture-laden sediment. This does not exclude that some initial dessication cracks may be present at the Vale de Meios tracksite. In any case, cracking due to dinosaur activity seems to be the number one cause of non-tectonic cracking.

### The Vale de Meios tracksite

**Figure 1:**
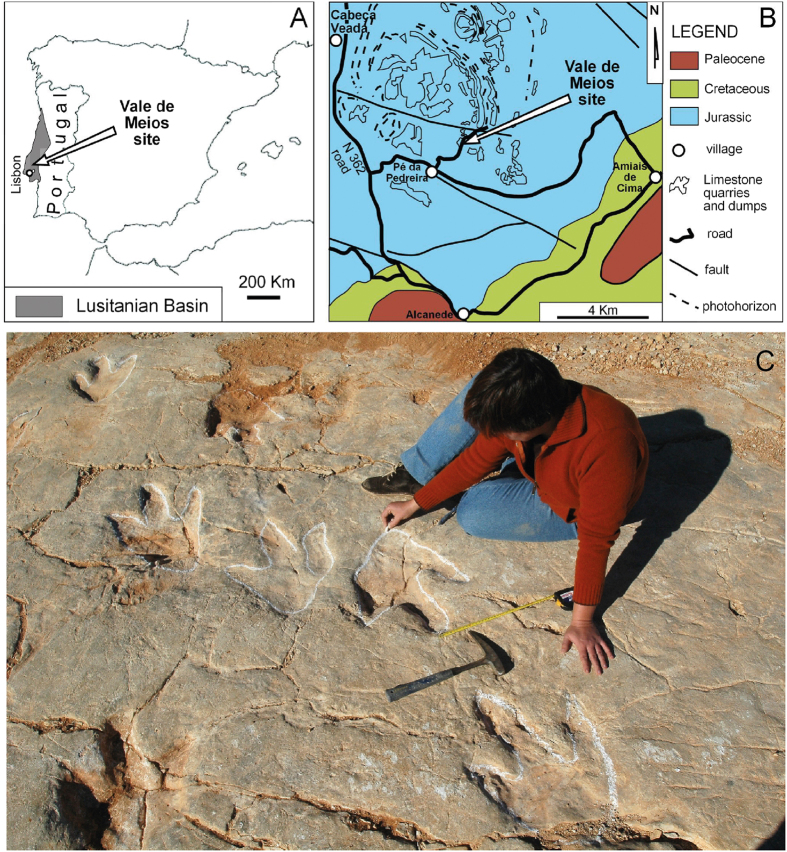
Geographical and geological setting of the Vale de Meios tracksite within the Lusitanian basin. (**A**) Outiline drawing of the Iberian Peninsula with location of Lusitanian Basin and the Vale de Meios site. Drawing originated through Adobe Illustrator CS5, version 15.1.0, www.adobe.com. (**B**) Compound of local geology and geography redrawn from Carvalho *et al*.^17^ and originated through Adobe Illustrator CS5, version 15.1.0, www.adobe.com. (**C**) Part of the tracking surface of the Vale de Meios site. (Original drawings by O.O. and original photo by Luis Quinta).

**Figure 2 f2:**
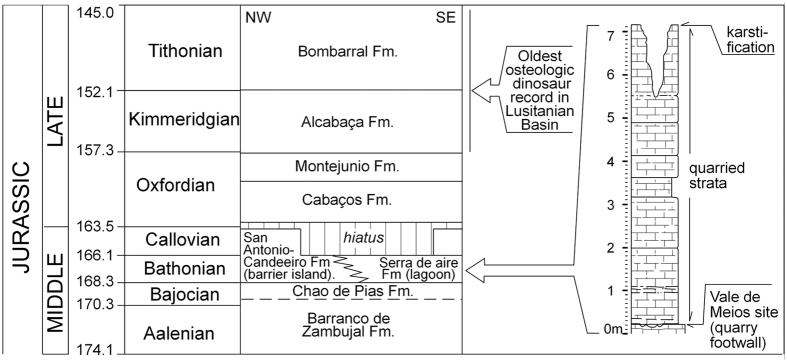
Local stratigraphy at Vale de Meios site and correlation with the stratigraphy of the Middle and Late Jurassic of the Maciço Calcareo Extremenho (Carvalho *et al*.^17^, center). Left: chronology *sensu* Grandstein *et al*.^65^.

**Figure 3:**
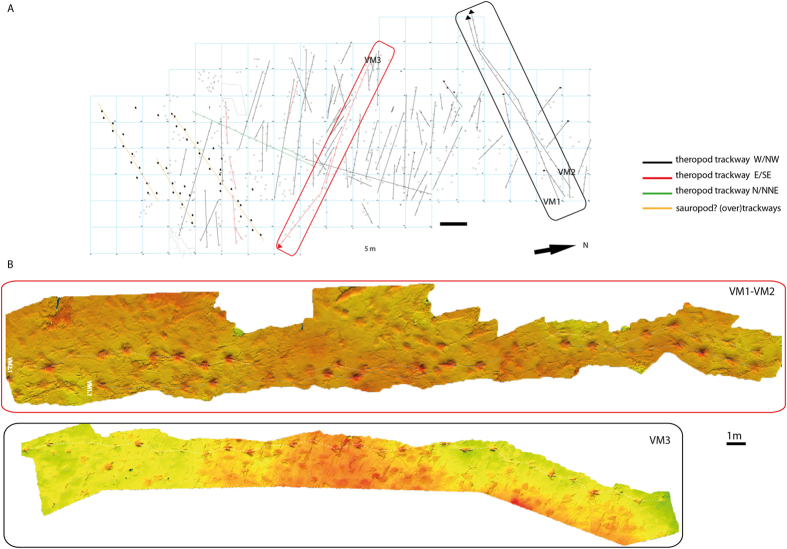
Cartography of the Vale de Meios site and photogrammetric models of three analysed trackways. (**A**) 2-D cartography of the Vale de Meios site, trackways directions indicated in the legend with different colours (black, red, green and orange). (**B**) 3-D photogrammetry models undertaken for three analysed trackways VM1, VM2 and VM3. See Supplementary Information for three-dimensional models visualization of trackways VM1, VM2 and VM3.

The Vale de Meios tracksite (Figs 1, 2, 3) was first discovered in 1998 by the technicians of the natural park of the Serra de Aire e Candeiros. Since its discovery, researchers of the National Museum of Natural History and Science (Lisbon, Portugal) presented preliminary evaluations on the site^13^,^16^,^25^. The locality, situated near Pé da Pedreira village (Alcanede, West-Central Portugal; 39°27'30.26“N, 8°49'11.07“W) has a total area of 7,500 m^2^ (Fig. 3). The area shown in the map is of 4,275 m^2^, with a total number of 711 recorded theropod tracks (but more than 3,000 estimated) organized in more than 80 trackways (Fig. 3A). The trackways are long (trackway lengths range from 30 to 40 meters) and show straight (unidirectional) paths with a bimodal orientation pattern. From the directional analyses we distinguished more than 10% of the trackways with an E/SE orientation while the majority of the trackways following the opposite W/NW orientation. There are some crossing areas between different trackways; most of them correspond to crossing trackways orientated in opposite directions. No evidence of trackways turning back or re-crossing themselves have been observed.

### Systematic paleontology

#### Megalosauripus isp

Material. trackways VM1 (24 tracks), VM2 (28 tracks) and VM3 (29 tracks), two isolated tracks (VMX.1, VMX.2 illustrated in Fig. 5A′B′) and trackways VM4-VM80 from the 2-D cartography map in black, red and green colour (Figs 3A and 5C–F).

Locality. Vale de Meios tracksite, Pé da Pedreira (village nearby), Alcanede, West-Central Portugal.

Horizon. Serra de Aire Formation (Bathonian).

Description. Tracks are tridactyl, sometimes tetradactyl (hallux impression, Figs 6I,K and 7H), large (TL range from 22 cm to more than 80 cm), elongated (TL/TW ranges from 1.24 to 1.39) and asymmetric. The mesaxonic index ranges from weak mesaxony, implying a short developement of digit III or a longer distance between digit impressions II-IV to a stronger mesaxony, with a long development of digit III or shorter distance between digit impressions II-IV (anterior triangle l/w ranges from 0.26 to 0.48, Fig. 5A–F). They are featured by the general absence of clear pad impressions, although they do display them in tracks VMX.1 and VMX.2 (Fig. 5A′‴ and Supplementary Information for three-dimensional models), the presence of pointed claw marks, a slightly sigmoidal impression of digit III and a squared U-shaped metatarso-phalangeal impression. Interdigital angles are variable along a trackway, with general low values for both II^III and III^IV (minimum 22° maximum 40°) reflecting a minor parallelism of digits on the distal anterior half of the track. The difference between interdigital angles II^III and III^IV is usually less than 10°. Pace length and pace angulation are very irregular (e.g. in trackway VM1, pace length SD ± 19.6, pace angulation SD ± 9.01, Table 1), with an inward rotation of the distal end of digit III impression with respect to the trackway middle line. Trackways VM1 (24 tracks) and VM2 (28 tracks) are directed toward W/NW and measure 35 and 40 meters respectively, while trackway VM3 (29 tracks) is directed toward E/SE and it measures 30 meters in total lengths. See Table 1 for full measurements, Table 2 for average measurements and Supplementary Information for three-dimensional models.

### Remarks

Tracks from the Vale de Meios tracksite are here compared with the main valid large theropod ichnotaxa regardless of the geography and time-period (Fig. 8). *Kayentapus*^26^ (Fig. 8A) do not fit into the studied tracks because of the smaller size, the higher TL/TW index, the wider width of the interdigital angles (considering variations) and the presence of diagnostic phalangeal pad formula, not consistently appreciable in Vale de Meios. Furthermore, TL/TW index in the studied tracks ranges from 1.24 to 1.40, differing greatly from that of *Grallator* (2.64 in Olsen *et al*.^27^) and *Eubrontes* (1.70 in Olsen *et al*.^27^; Fig. 8B). The AT l/w relationship for *Eubrontes* (0.58; Lockley^15^) and *Grallator* (1.22; Lockley^15^) display a much stronger mesaxony than the Vale de Meios tracks (from 0.26 to 0.48). Though, *Eubrontes* type tracks are of significantly varied morphologies in Jurassic and Lower Cretaceous formations in China, such as generally low TL/TW like 1.4 in Hanxi tracksite^28^. *Irenesauripus*^29^ (Fig. 8C) from the Aptian–Albian of Canada strongly differs with the Vale de Meios tracks in the very narrow and slender digits and the larger interdigital angle. Besides some similarities in size and proportions of the 86-cm-long *Tyrannosauripus pilmorei* track^30^ (Fig. 8D) and the recently erected new ichnogenus and ichnospecies *Bellatoripes fredlundi*^31^ (Fig. 8E) from the Upper Cretaceous of North America, they differ from the Vale de Meios tracks especially on the robustness of the digit impressions, which are proximally wide and strongly taper distally, on the lack of a clear phalangeal pad formula and in wider metatarsal pad trace. The emended *Bueckeburgichnus maximus* track^32^ (Fig. 8F) from the Lower Cretaceous of Germany is similar to the Vale de Meios tracks in size (TL: 56 cm) and in the medially-directed hallux impression, but they clearly differ in the presence of a more massive metatarsal area, in the lateral digits broadness and divergence of digit IV and in the longer digit III impression resulting in a stronger mesaxony (> 0.55)^33^. *Eutynichnium lusitanicum*^34^ (Fig. 8G) is another large theropod described from the Late Jurassic of Portugal and diagnosed on the presence of an anteriorly oriented hallux, short metarsal and stocky and non taper digits impressions. Nontheless, in the few tetradactyl tracks preserved in the Vale de Meios tracksite, the hallux is medially oriented (Fig. 6I,K), the metatarsal is elongated (Fig. 7H) and digit impressions are slender and taper.

The Vale de Meios tracks encompass *Iberosauripus grandis*^33^ (Tithonian-Berriasian, Spain; Fig. 8H) in their minimum values for the TL/TW ratio (1.30; Vale de Meios: 1.24–1.40), AT l/w relationship (0.30; Vale de Meios: 0.26–0.48) and interdigital angles II^III and III^IV (<20°; Vale de Meios: >20°). The main morphological differences noticed are the broadness of the toes, the pad presence and the general symmetry of *Iberosauripus grandis*.

The Vale de Meios tracks display similar values with *Megalosauripus uzbekistanicus* (Fig. 8I) for the TL/TW ratio (1.21 in Fanti *et al*.^35^), the interdigital angles are 40° (II^III) and 30° (III^IV) and the AT l/w relationship (0.40 reported in Cobos *et al*.^33^). Furthermore, similar morphological features that *M. uzbekistanicus* shares with the Vale de Meios tracks are the sigmoidal impression of digit III, the presence of hallux (although it is not strictly an ungueal impression *sensu* Fanti *et al*.^35^ in the Portuguese tracksite) and the shape of the phalangeal-metatarsal pad impression as observed in Fig. 7B of Fanti *et al*.^35^. The morphology of Middle Jurassic *Megalosauripus*-like tracks from the Cleveland basin^12^ (Fig. 8H) and the Ardley Quarry^11^ (Fig. 8I) is also very similar to the Vale de Meios tracks in the inward rotation of digit III, the moderate divergence of the weight-bearing toes (II-IV), the average TL/TW index (1.40). Furthermore, Late Jurassic *Megalosauripus*-like morphotypes recognized in Arizona and Utah (Fig. 8L,M; Lockley *et al*.^34^) and Morocco^36^ (Fig. 8N) also recall the studied track morphologies.

For similarities with both qualitative and morphometric parameters of *Megalosauripus uzbekistanicus* together with the strong resemblance with the aforementioned *Megalosauripus*-like tracks, the Vale de Meios tracks are here assigned to *Megalosauripus* ichnogenus, representing the oldest occurrence of this ichnotaxon.

The assignment to *Megalosauripus* isp. is based on general morphology and morphometric ratios, irrespective of differences in the track lengths. Therefore, the intra-trackway track length variation discards the possibility that the site was crossed by a stock of taxonomically diverse theropods. This is the reason explaining that the track morphology remains the same among tracks with different sizes. As a result, isolated small-sized tracks could be the reflection of a high variety of preservational modes (due to different stages of substrate consistencies) or to different ontogenetic stages of the trackmakers. Finally, preservation of tracks could be strongly influenced by the tidal cycle, which produced preservations types such as modified true tracks and modified true tracks with mud collapsing through erosion and water saturation respectively.

## Tracks preservation

Only few tracks are considered well-preserved while the rest are morphologycally affected by substrate consistency changes or taphonomical processes transforming true tracks with anatomical details and preservation grade between 2 and 3 (following Belvedere and Farlow^37^), into different gradients of modified true tracks according to water content, erosive factors (Fig. 6), primary features (i.e. radial fractures) and secondary features (i.e. internal overtrack formation, Fig. 7). Throughout the outcrop, no clear spatial distribution of these preservational types is observed. Tracks display three different types of taphonomic preservations:

1. *True tracks with preservation grade between 2 and 3* (Fig. 6A–D). Following Belvedere and Farlow^37^, this type of tracks is comprehended between grade 2, in which tracks preserve fairly clear and sharp toe marks, ungual marks and some digital pads recognizable and grade 3, in which all digit impressions are completely sharp and clear, digit walls well defined, ungual marks and distinct digital pads clearly preserved. As a result of the environmental setting, characterized by moist sediment, these types of tracks are not so common at the Vale de Meios tracksite (5%).
2. *Modified true tracks* (Fig. 6E–H). This type of preservation, as described in Marty^21^, is modified by physico-chemical (e.g.,weathering) and/or biological influences after they were made. It is the most abundant type of the site (75%), as it could be expected by the non-laminated nature of the tracking surface. Note that this preservation represents modified true tracks in the sense of Marty^20^ and Marty *et al*.^38^, that is to say, the track is not morphologically overestimated due to depth propagation.
3. *Modified true tracks with mud collapsing* (Fig. 6I,L). These tracks result from water-saturated sediments and are evidenced by the collapse of the sediment inside the digits and ocasional metarsal and hallux impressions. It is remarkable that throughout VM1, VM2 and VM3 trackways, the degree of mud collapsing is variable, causing intra-trackway track length variability (*sensu* Razzolini *et al*.^39^).

Preservation of tracks could be strongly influenced by the tidal cycle, which produced preservation grades such as modified true tracks and modified true tracks with mud collapsing through erosion and water saturation respectively.

All three preservation types can display two associated features: radial fractures and internal overtracks (Fig. 7). Radial fractures have been described in literature of general and experimental ichnology^20^,^38^,^40^,^41^. In the Vale de Meios tracksite, radial fractures are found in most of the tracks (Fig. 6 and 7A–D), are always normal to the profile of the print and develop preferentially from the claws outwards. Typically, more than 10 fractures per track are observed and they may branch out. They reach a longitude of up to 50 cm and the width of the open space is variable, but generally less than 0.5 cm. These structures are not strictly linked to the ocurrence of the displacement rims as it happens in other cases (Fig. 5E in Marty *et al*.^38^). Other longer (centimeters to tens of meters) non-radial fractures are also observed (Fig. 7E).

**Figure 4:**
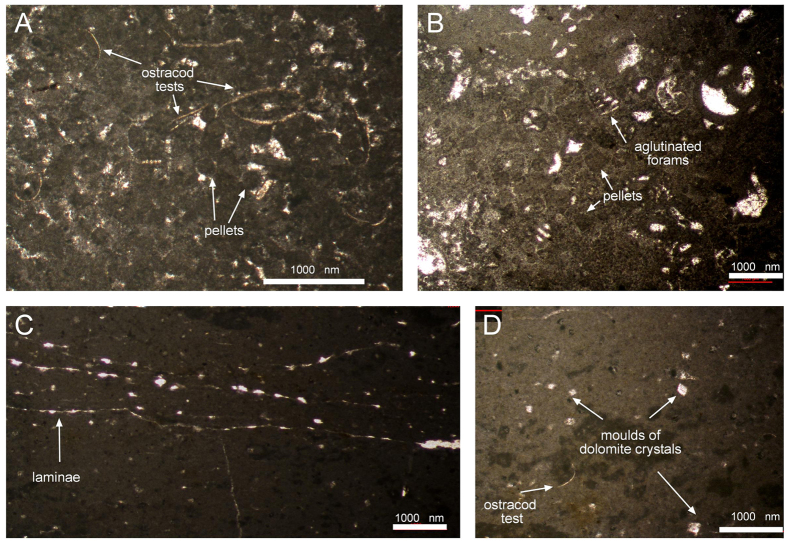
Thin sections of sediment samples IPS87258, IPS87264 and IPS87259 collected both on track surface level and tracks overfill. (**A,B**) massive limestone, (**B,C**) laminated limestone. Scale bar 1000 nm.

**Figure 5:**
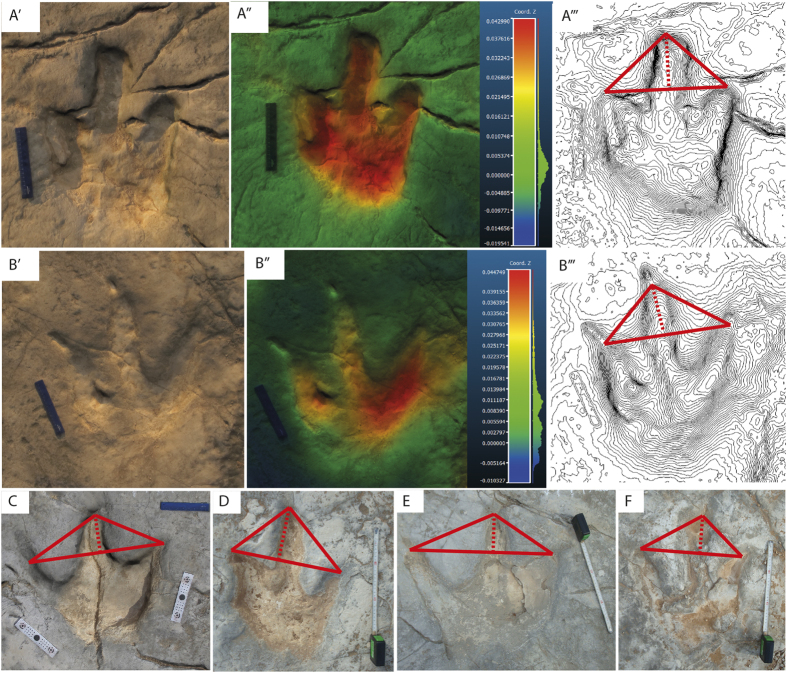
Morphometric comparison among track morphologies in the Vale de Meios tracksite. Triangles are drawn following Lockley^15^, showing the index of mesaxony with the anterior triangle l/w relationship (AT l/w). A‴ track VMX.1, 0.462, B‴ track VMX.2, 0.351 (**C**) 0.278, (**D**) 0.486, (**E**) 0.267, (**F**) 0.368. Scale bar in (**A–C**), 15 cm; scale bar in (**D–F**) 30 cm. See supplementary Information for three-dimensional models visualization of tracks VMX.1 and VMX.2.

Regarding internal overtracks (Fig. 7E–H, *sensu* Marty^20^) they are very common and can also be found in all the three preservation types. Probably, the lack of this feature in some tracks is the result of recent removal during quarry works. A remarkable feature is that overtrack sediment wedges towards the edges of the track. The samples collected (Fig. 4C,D) revealed that the thin lamination of the sediment inside the track is due to microbial mats. The track bottom (true track *sensu stricto*) was covered with water during tidal events and the resulting internal overtrack was induced by repeated growth of microbial mats in the wetter track interior, by the trapping of sediment, or by an alternation of both processes. After the track formation, microbial mats developed preferentially within the tracks, as observed by the internal overtracks (Fig. 7E–H). This kind of overtracks has been commonly reported in other tidal environments^21^,^38^,^42^.

The relationship between tracks and associated features do not only provide a cross cutting sequence, but also clues to the origin of fractures. Non-radial fractures are tectonic joints, as supported by their length (up to tens of meters) and by the parallel disposition in joint families. Sometimes, non-radial fractures have calcite crystals infill. Additionally, non-radial fractures crosscut both the tracking surface and internal overtracks. In contrast, radial fractures never cut the internal overtrack, i.e. radial cracking is previous to the internal overtrack formation.

## Trackmaker identification

The Vale de Meios trackmakers are large theropods or megatheropods as their estimated hip heights overpass the threshold (250 cm) proposed by some authors^33^,^43^ and the footprint length exceed 45 cm^20^,^43^,^44^. These theropod tracks are among the largest theropod tracks described worldwide^30^,^31^,^45^,^46^. Nevertheless, other very large tracks are known. In general, trackmaker identity should reflect the least inclusive group that bounds all taxa sharing similar morphological characteristcs and spatiotemporal distributions. Therefore, in order to ascertain which group of theropods might be the best trackmaker candidate for the studied tracks, we reviewed the bone record of large-sized theropods in the Middle Jurassic of Europe. In the Iberian Peninsula, the osteological remains for this clade at that age are absent; out of this region, theropod osteological remains are recovered mainly from England (*Duriavenator hesperis*^47^; *Megalosaurus bucklandii*^6^, *Magnosaurus nethercombensis*^7^; *Cruixicheiros newmanorum*^8^), France (*Poekilopleuron bucklandii*^4^,^5^; *Dubreillosaurus valesdunensis*^3^). They are all Bajocian-Bathonian in age and have been attributed to the Megalosauridae family, which is the dominant clade for the Middle Jurassic in Europe.

The synapomorphy-based correlation of the trackmakers depends on appendicular and pedal elements, which are usually lost during fossilization^48^. Plus, the osteological convergence and substantial overlap in phalangeal proportions of the theropod foot would not allow a lower level distinction among different theropod taxa^48^. Buckley *et al*.^49^ indicate that tracks are not consistently preserved so as to reproduce the proportions of the trackmaker’s foot with perfect fidelity, especially during animal locomotion. However, considering additional data such as the size and the provenance (taking into account both temporal and spatial distributions)^48^, there are no other possible candidates other than megalosaurids, as this is the unique group of large theropods capable to produce large tracks during the Bajocian-Bathonian times.

## Megalosaurid behaviour inferred from tracks

The orientation patterns of the trackways can provide useful information about the behaviour and habitat propensity of the trackmakers, especially if there is some preferred orientation of the trackways^50^,^51^. For example, Day *et al*.^11^ reported various trackways at the Ardley Quarry, a Middle Jurassic tracksite with similar theropod tracks and trackways. The Ardley Quarry trackways display a degree of parallelism, suggesting that the trackmakers movements were either constrained by a linear geographical feature, or that they were moving in a herd. Generally, unidirectional orientation patterns, together with other parameters (similar locomotion velocity, regular intertrackway spacing, identical pace rhythm) are the best evidence to suggest gregarious behaviour among the trackmakers^31^,^52^. It is noteworthy that this kind of behaviour is not usually reported in large theropods on the basis of the footprint record^53^,^54^,^55^,^56^. Moreover, the presence of a huge number of large theropod footprints (more than 700 hundred) is highly uncommon in the fossil record and the Vale de Meios tracksite is therefore a rare site of great paleobiological and paleoethological relevance.

The detailed picture of the Vale de Meios tracksite shows an inter-tidal flat crossed normally by large theropods showing a general bimodal orientation pattern. The tidal flat is located in an inner platform (i.e. landwards edge of a lagoon, Fig. 9A) with a coastal barrier arranged in a E/NE-W/SW orientation. The majority of trackways (black colour, Fig. 3A) follows a W/NW orientation, toward the barrier (Fig. 9B). In contrast, the E/SE direction of trackways (red colour, Fig. 3A) is directed opposite, towards the land edge of the inner platform.

Bimodal orientation patterns have often been associated with physical features of the paleoenvironment such as the shoreline^57^,^58^ and also to the paleogeographic conditions^59^. For instance, the most common condition found in fossil and modern trackways is that of trackways running parallel to the shoreline, typically linked to migratory animals moving from one area to another within the lake^51^. Besides, these authors suggested “shoreline position exerts a stronger influence on the distribution of animal activity than any other environmental factor”.

**Figure 6:**
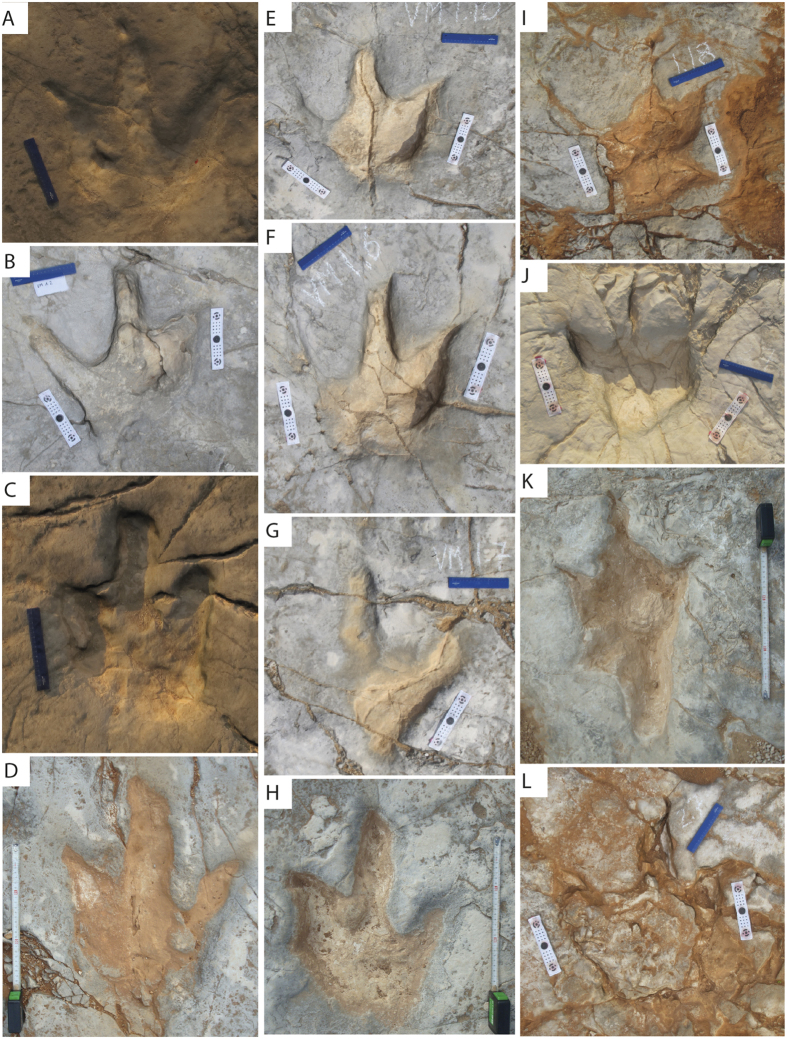
Different preservations observed in the Vale de Meios tracksite. (**A–D**) True tracks with degree of preservations between 2 and 3 (following Belvedere and Farlow^37^). (**H,I**) modified true tracks preservation. (**I,L**) true tracks with mud collapsing. This type of preservation of tracks accounts for the 5%, 75% and 20%, respectively in the whole tracksite.

**Figure 7:**
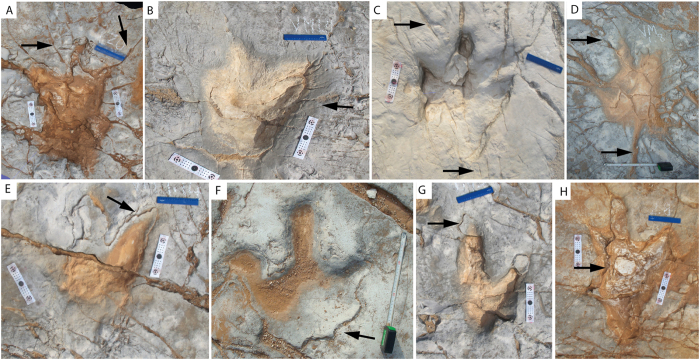
All types of preservations can display two associated features. (**A–D**) radial fractures; (**E–H**) internal overtrack (*sensu* Marty^20^).

**Figure 8:**
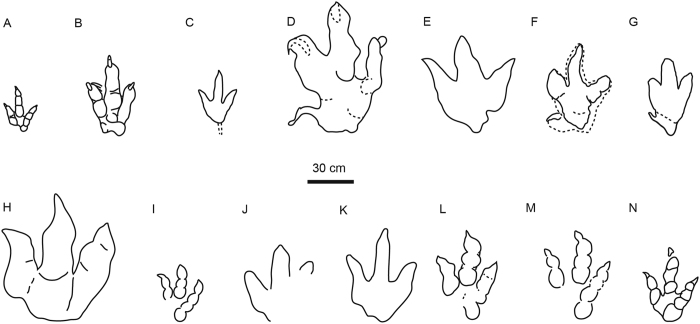
Redrawn outlines of the main large theropod ichnotaxa all to scale (30 cm). Left tracks are mirrored as right footprints. Drawing originated through Adobe Illustrator CS5, version 15.1.0, www.adobe.com. (**A**) *Kayentapus*^26^; (**B**) *Eubrontes*^27^, (**C**) *Irenesauripus*^29^, (**D**) *Tyrannosauripus pillmorei*^30^, (**E**) *Bellatoripes fredlundi*^31^, (**F**) *Bueckeburgichnus maximus*^32^, (**G**) *Euthynichnium lusitanicum*^34^, (**H**) *Iberosauripus grandis*^33^, (**I**) *Megalosauripus uzbekistanicus*^35^ (**J**) *Megalosauripus*-like^13^, (**K**) *Megalosauripus*-like^12^, (**L**) *Megalosauripus* from Arizona^34^, (**M**) *Megalosauripus* from Utah (*sensu* Lockley *et al*.^34^), (**N**) *Megalosauripus*-like from Morocco^36^ (All drawings redrawn by NLR).

**Figure 9:**
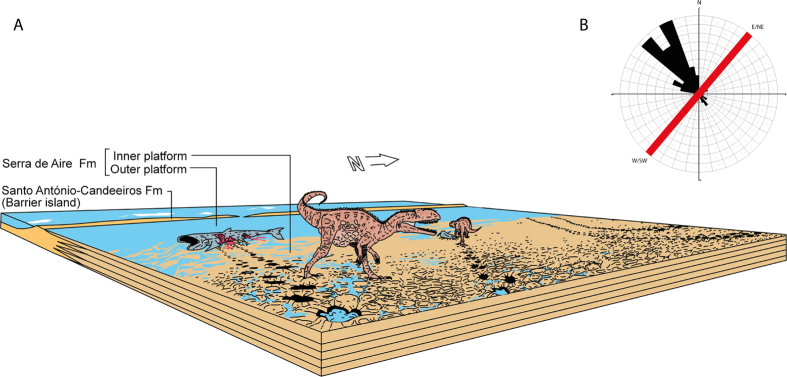
Paleoenvironmental and paleoethological reconstruction of the tidal flat crossed by megalosaurids feeding on exposed carcasses during low tides. Original drawing by Oriol Oms, originated through Adobe Illustrator CS5, version 15.1.0, www.adobe.com. (**A**) Orientation of the coastal barrier extrapolated from Azeredo *et al*.^18^ (**B**) Rose diagram with directions of trackways resulted in a unidirectional bimodal orientation, normal to the coastal barrier one. Red line is the orientation of the barrier island.

Nevertheless, the opposite trend is observed for the Vale de Meios trackways where the bimodal orientation pattern is represented by trackways (the majority of them) directed perpendicular to the shoreline. In fact, 90% of the trackways is subparallel and are heading to the barrier while 10% of the trackways is heading opposite to the barrier (E/SE direction). Cohen *et al*.^51^ also reported perpendicular trackways to the shoreline suggesting that animals can approach the margin of the lake to “drink, forage, or pass by (or, in the case of carnivores, to hunt herbivores doing any of the above)”. Following Getty *et al*.^56^, if the subparallel orientation of the trackways is not caused by the gregarious behaviour, something else must have caused it. It should be noted that the parameters suggesting gregarious behaviour are not fully appreciable for the Vale de Meios trackways. Anyway, what seems clear is that the bimodal orientation pattern in the case of Vale de Meios is not related to the shoreline configuration as in the aforementioned papers. The sedimentological and taphonomic analyses together with data on the distribution and orientation of trackways permit us to infer theropod behavior throughout the tidal flat environment. Thus, the majority of trackways at Vale de Meios is likely to have been impressed during low tide periods, when the conditions to produce footprints are more suitable. The new surfaces exposed during the low tide periods favoured the preservation of footprints and the moisture-laden sediment counts for the variety of preservation modes (Fig. 6). A possible explanation for the direction of movement of the majority of trackways (black colour, Fig. 3A) is that of megalosaurids crossing the exposed area of the tidal flat when the water recides, that is to say during low tide periods. This hypothesis is based on the strong directionality (and bimodality) in theropod paths, normal to the barrier. The long linear trackways across the site represent a directional pattern (*sensu* Cohen *et al*.^51^) suggesting that the megalosaurids cross the tidal flat with a precise purpose (not milling).

The unusual behaviour of large theropods moving toward the coast had not been previously documented and entails the possibility that megalosaurids invaded the area to feed on fish, invertebrates and other vertebrates exposed on the tidal surface. Although there are examples in literature of gregarious behaviour in large theropods supported through both bonebeds^60^ and trackways^31^, it has been usually suggested that large theropods were solitary hunters^61^,^62^ The numerous trackways might represent few individuals crossing the tidal flat recurrentely. In fact, some reports of theropods moving towards and away from the shoreline have been considered possible evidence of piscivory^58^ or feeding on other vertebrate carcasses (*sensu* Roach and Brinnkman^63^ and *contra* Ostrom^53^).

The inferred piscivory diet of megalosaurids is not unexpected and has been documented by stomach contents in *Poekilopleuron*^64^. Allain^3^ stated that the inclusion of fishes as part of the megalosaurid diet is consistent with both taphonomic and phylogenetic data. Moreover, the deposits yielding the described megalosaurid taxa indicate paralic and shallow marine environments, including marine-influenced lagoon^9^ and coastal mangrooves grounds^3^. These data combined with the trackway evidence from Vale de Meios may suggest that megalosaurids frequented this palaeonvironment, and similar to spinosaurids, would have been opportunistic carnivores, feeding on terrestrial vertebrates but also on fishes. In this regard, the long trackways documented at Vale de Meios tracksite reveal a stock of large megalosaurids moving to the shoreline and back from the land to the coastal barrier and invading new exposed areas of the tidal flat. The reason of such striking behaviour could be the occasional piscivory diet of megalosaurids, as these large theropods would take advantage of new exposed areas to feed on fishes and other vertebrates.

## Conclusion

The Vale de Meios limestone quarry from the Serra de Aire Formation, Bathonian in age (Santarém, West-Central Portugal) is a key and unique reference for understanding the composition and distribution of the Middle Jurassic theropod fauna, especially due to both the ichnological and osteological record for this age being extremely scattered. In this study, tracks and trackways from the whole tracksite are assigned to *Megalosauripus* isp. according to quantitative and qualitative analyses and comparisons undertaken. This ichnogenus occurrence, traditionally reported for the Late Jurassic-Early Cretaceous, should therefore be expanded also to the Middle Jurassic. The Vale de Meios tracks are among the largest theropod tracks ever reported, and they were produced by large individuals of the Megalosauridae family, the dominant tetanuran clade during this age in Europe. Furthermore, this is the first tracksite in which *Megalosauripus* is in a probable coincident correlation with megalosaurids. The directional analyses of trackways, which are preserved in an inter-tidal flat located at the edge of a lagoon, reveals that various individuals crossed a tidal flat in accordance to tide cycles, directing toward the barrier during low tide periods, probably for feeding purposes on exposed vertebrate. Such clear bimodal orientation arrangement (forth and back) interpreted as single or small aggregates of large theropods individually moving toward a carcass on the shoreline is highly uncommon as it is the presence of such a large number of large theropod footprints.

## Additional Information

**How to cite this article**: Razzolini, N. L. *et al*. Ichnological evidence of Megalosaurid Dinosaurs Crossing Middle Jurassic Tidal Flats. *Sci. Rep*. **6**, 31494; doi: 10.1038/srep31494 (2016).

## Supplementary Material

Supplementary Information

## Figures and Tables

**Table 1:** Complete measurements in centimeters (TL, SL, PL), angles (PANG) and ratio indexes (TL/TW and AT l/w) from trackways VM1, VM2 and VM3.

TRACKWAY	TL	TW	TL/TW	PL	SL	P.ANG
VM1.1	59,5	54,7	1,1		297,9	161
VM1.2	60,6	54,2	1,1	134,2	321,8	168
VM1.3	61,2	53,6	1,1	163,5	330,3	165
VM1.4	59,1	51,6	1,1	161,1	316,0	164
VM1.5	54,3	42,5	1,3	165,5	313,9	157
VM1.6	51,2	40,9	1,3	158,9	322,9	158
VM1.7	58,4	54,3	1,1	165,6	322,6	160
VM1.8	60,2	51,6	1,2	156,7	324,1	151
VM1.9	60,6	53,1	1,1	181,3	297,7	153
VM1.10	52,2	46,9	1,1	165,5	304,1	138
VM1.11	59,7	50,1	1,2	157,6	367,3	155
VM1.12	63,2	45,8	1,4	178,2	387,5	161
VM1.13	100,8	50,5	2,0	225,1	333,2	149
VM1.14	66,6	45,8	1,5	175,2		175
VM1.15	64,9	52,6	1,2	169,6	323,2	
VM1.16						
VM1.17	60,5	56,3	1,1			
VM1.18	57,7	50,5	1,1	153,0	311,3	
VM1.19	56,5	54,6	1,0	163,2	305,3	
VM1.20	68,8	52,4	1,3	151,7	342,2	
VM1.21	65,4	55,7	1,2	192,6	313,2	
VM1.22	61,1	54,4	1,1	138,1	293,2	
VM1.23	83,9	51,8	1,6	167,0	308,8	
VM1.24	69,1	50,5	1,4	141,0		
**AVERAGE**	**63,3**	**51,1**	**1,2**	**165,0**	**321,8**	**158**
**SD**	**10,6**	**4,1**	**0,2**	**19,6**	**23,0**	**9,01466073**
**SPEED**				**1,541 m/s**	**5,547 Km/h**	
VM2.1	60,1	47,1	1,3		233,3	128
VM2.2	58,2	54,5	1,1	135,4	255,4	139
VM2.3	61,7	44,7	1,4	120,8	306,8	142
VM2.4	74,6	50,6	1,5	155,7	300,3	171
VM2.5	83,4	52,7	1,6	159,3	295,6	150
VM2.6	73,7	63,9	1,2	145,5	296,4	169
VM2.7	63,2	62,1	1,0	167,5	287,0	160
VM2.8	68,0	52,2	1,3	141,1	278,2	144
VM2.9	60,6	47,3	1,3	158,9	285,2	149
VM2.10	54,1	49,8	1,1	155,4	305,8	154
VM2.11	72,5	59,8	1,2	147,0	307,0	150
VM2.12	76,2	65,3	1,2	165,3	309,2	154
VM2.13	79,9	61,3	1,3	156,0	306,0	155
VM2.14	72,3	58,9	1,2	161,3	303,5	156
VM2.15	80,6	46,3	1,7	148,7	317,0	126
VM2.16	75,3	60,6	1,2	157,8		135
VM2.17	65,3	59,8	1,1	169,9		165
VM2.18						166
VM2.19						
VM2.20	84,6	48,7	1,7			
VM2.21	65,1	48,6	1,3	177,2		
VM2.22	81,6	56,5	1,4	155,6	322,6	
VM2.23	72,8	56,7	1,3	170,1	306,8	
VM2.24	74,2	57,6	1,3	200,6	332,9	
VM2.25	76,5	63,8	1,2	169,3	365,7	
VM2.26	69,0	48,4	1,4	143,2	294,0	
VM2.27	82,1	61,5	1,3	210,8	323,5	
VM2.28	80,4	57,6	1,4	163,0	348,7	
**AVERAGE**	**71,8**	**55,2**	**1,3**	**159,8**	**303,7**	**151**
**SD**	**8,6**	**6,3**	**0,2**	**19,0**	**28,0**	**13,2465651**
**SPEED**				**1,145 m/s**	**4,121 Km/h**	
VM3.1	37,8	25,3	1,5			
VM3.2				99,3	183,9	
VM3.3						
VM3.4	36,6	27,1	1,4			159
VM3.5	32,7	22,1	1,5	92,2		147
VM3.6	35,9	29,1	1,2	90,5	181,5	148
VM3.7	35,7	24,8	1,4	96,4	172,9	152
VM3.8	35,1	30,1	1,2	94,0	184,5	152
VM3.9	33,7	29,8	1,1	99,4	193,1	150
VM3.10	39,2	31,8	1,2	95,0	181,4	165
VM3.11	38,7	32,0	1,2	96,0	183,6	171
VM3.12	37,0	24,0	1,5	86,6	173,6	147
VM3.13	38,8	31,3	1,2	100,3	179,4	145
VM3.14	35,6	34,7	1,0	94,9	183,5	148
VM3.15	40,7	32,0	1,3	92,6	177,3	149
VM3.16	40,1	29,0	1,4	101,7	182,9	161
VM3.17	37,6	34,7	1,1	98,1	185,8	136
VM3.18	45,9	26,3	1,7	92,7	180,4	133
VM3.19	38,6	22,3	1,7	176,9	160,8	152
VM3.20	52,9	14,5	3,6	92,4	195,0	130
VM3.21	34,2	28,2	1,2	114,3	189,1	149
VM3.22	29,1	27,1	1,1	81,2	168,6	159
VM3.23	36,5	32,2	1,1	91,2	186,0	158
VM3.24	38,9	36,6	1,1	91,1	187,9	152
VM3.25	32,6	28,5	1,1	94,0	183,6	140
VM3.26	39,6	34,0	1,2	96,1	186,5	153
VM3.27	38,5	29,0	1,3	92,6	191,6	151
VM3.28	41,8	26,1	1,6	98,6	182,5	
VM3.29	49,5	34,4	1,4	97,4		
**AVERAGE**	**38,3**	**28,8**	**1,4**	**98,3**	**182,3**	**150**
**SD**	**5,0**	**4,8**	**0,5**	**17,1**	**7,7**	**9**
**SPEED**			**1,022 m/s**	**3,679 Km/h**		

Speed equation following Alexander^66^ formula V = 0.25 g^0.5*SL^1.67*H^−1.17.

**Table 2:** Average measurements in centimeters (TL, SL, PL), angles (PANG) and ratio indexes (TL/TW and AT l/w) from trackways VM1, VM2 and VM3.

TRACKWAYS	TL	TL/TW	AT l/w	SL	PL	PANG
**VM1**	63.28	1.24	0.46–0.48	321.81	164.97	158°
**VM2**	71.77	1.31	0.40–0.48	303.66	159.79	151°
**VM3**	38.27	1.39	0.26–0.27	182.30	98.29	150°
