# Supplementary material for: Ichnological evidence of Megalosaurid Dinosaurs Crossing Middle Jurassic Tidal Flats
Source: Sci Rep. 2016 Aug 19;6:31494. doi: 10.1038/srep31494 (PMC4990902; doi:10.1038/srep31494)
Supplement: Supplementary Information [file srep31494-s1.doc]

**Ichnological evidence of Megalosaurid Dinosaurs Crossing Middle Jurassic Tidal Flats**

Novella L. Razzolini^1*^, Oriol Oms^2^, Diego Castanera^3^, Bernat Vila^1,4^, Vanda Faria dos Santos^5^, Àngel Galobart^1,4^

^1^: Mesozoic Research Group, Institut Català de Paleontologia 'Miquel Crusafont', C/ Escola Industrial 23, 08201 Sabadell, Catalonia, Spain

^*^ Correspondence to novella.razzolini@icp.cat

^2^:Universitat Autonoma de Barcelona, Facultat de Ciències (Geologia), 08193, Bellaterra (Spain)

^3^: Bayerische Staatssammlung für Paläontologie und Geologie and GeoBioCenter, Ludwig-Maximilians-Universität, Richard-Wagner-Str. 10, 80333 Munich, Germany.

^4^: Museu de la Conca Dellà, carrer del Museu, 4, 25650, Catalonia, Spain.

^5^: Museu Nacional de História Natural e da Ciência – Universidade de Lisboa, Rua da Escola Politécnica, 58, 1250−102 Lisboa, Portugal

**
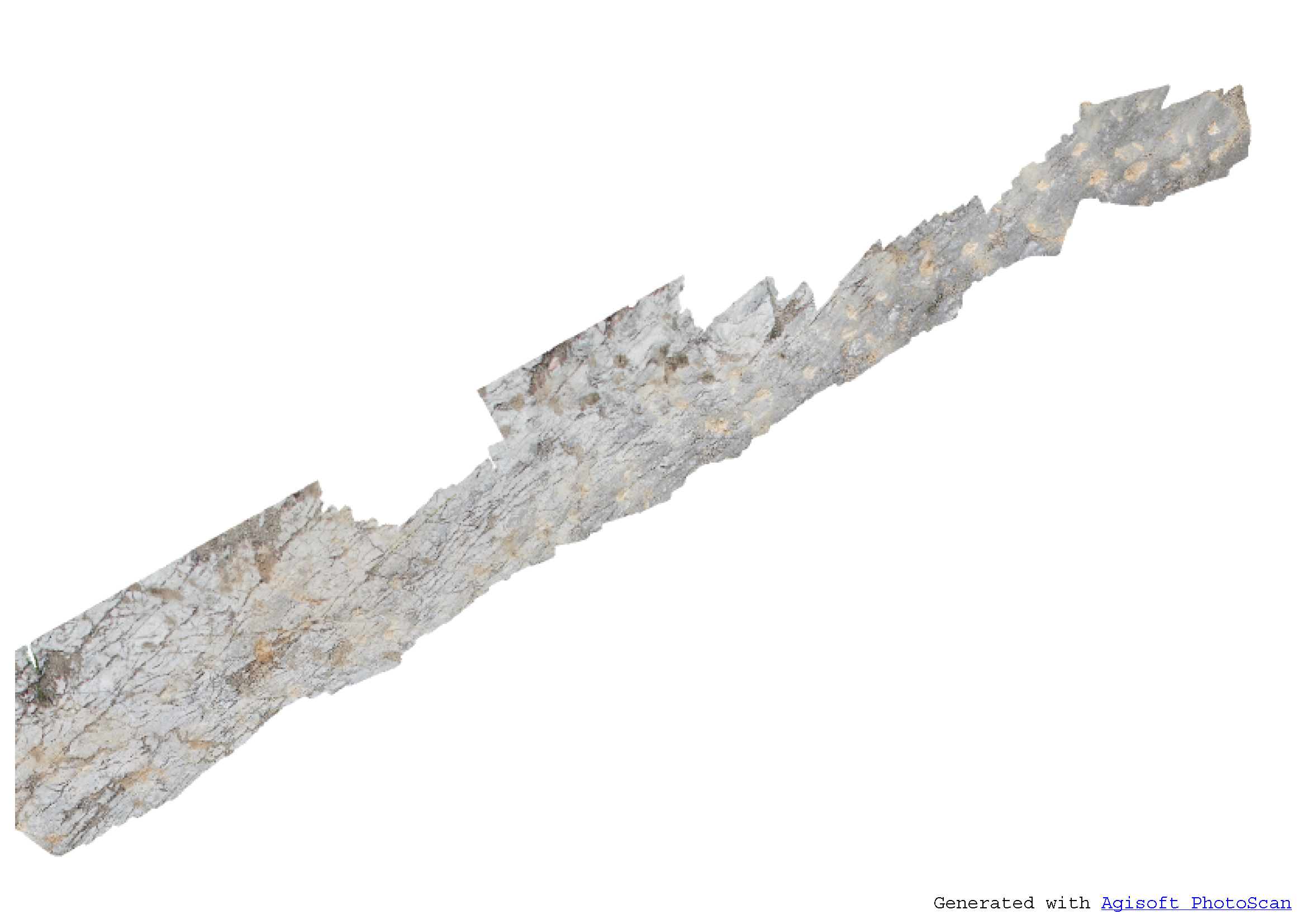
**

**Supplementary Data 1**. Digital reconstruction of trackways VM1 (24 tracks) and VM2 (28 tracks) directed toward W/NW and measure 35 and 40 meters respectively. Downloadable, interactive 3D PDF file and 3D model in .ply format generated through Agisoft Photoscan software available here: <https://figshare.com/s/4a0cc7cd871e17b0c3d2>,

10.6084/m9.figshare.3198673.

(Note: all 3D PDF files and 3D models, Supplementary Data 1-4, may be downloaded here: <https://figshare.com/s/2224473dc15a8c17a3d4>)


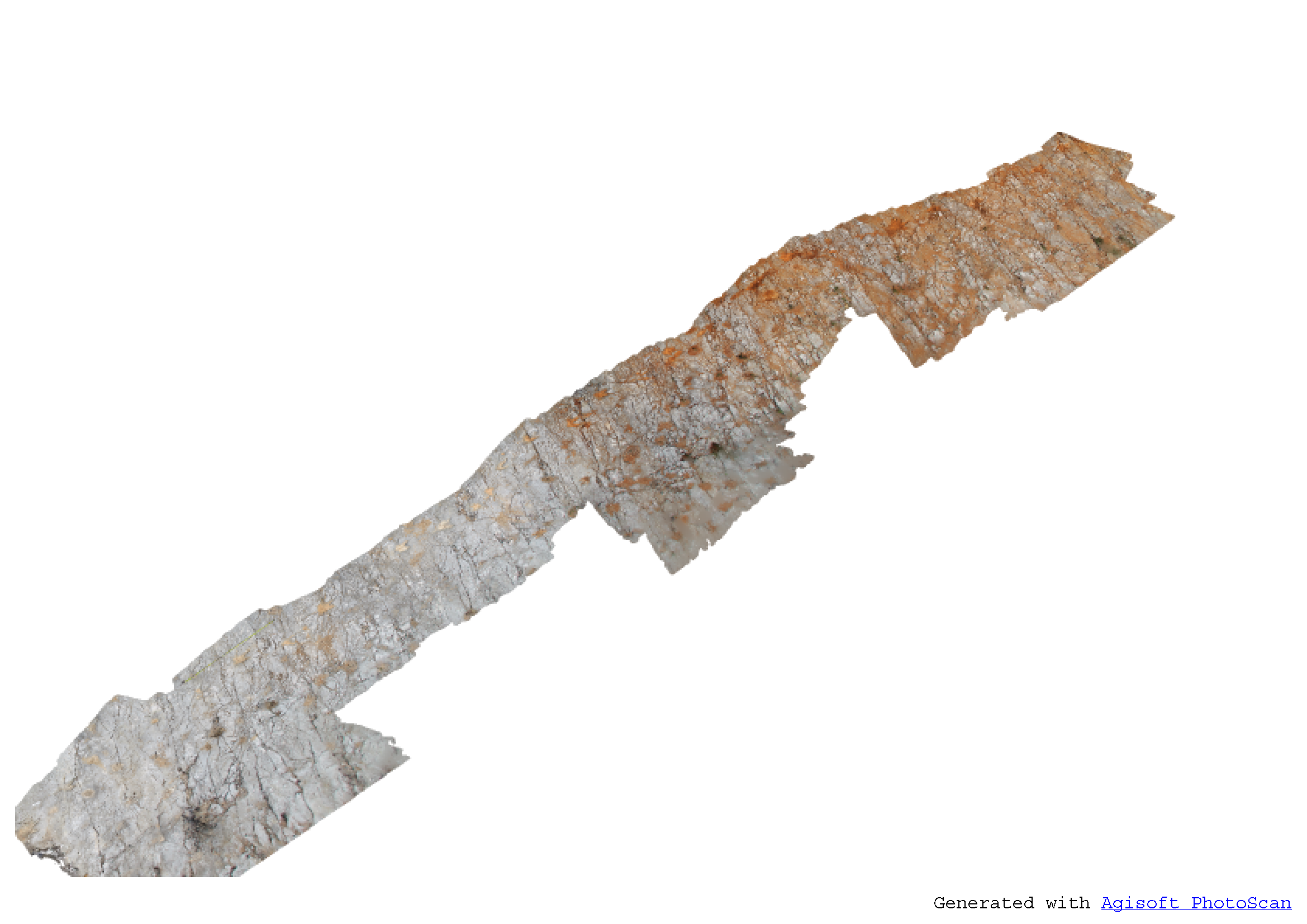


**Supplementary Data 2.** Digital reconstruction of trackways VM3 (29 tracks) directed toward E/SE and it measures 30 meters in total length. Downloadable, interactive 3D PDF file and 3D model in .ply format generated through Agisoft Photoscan software available here:

<https://figshare.com/s/412e5c47d578b363fb48>

10.6084/m9.figshare.3405763


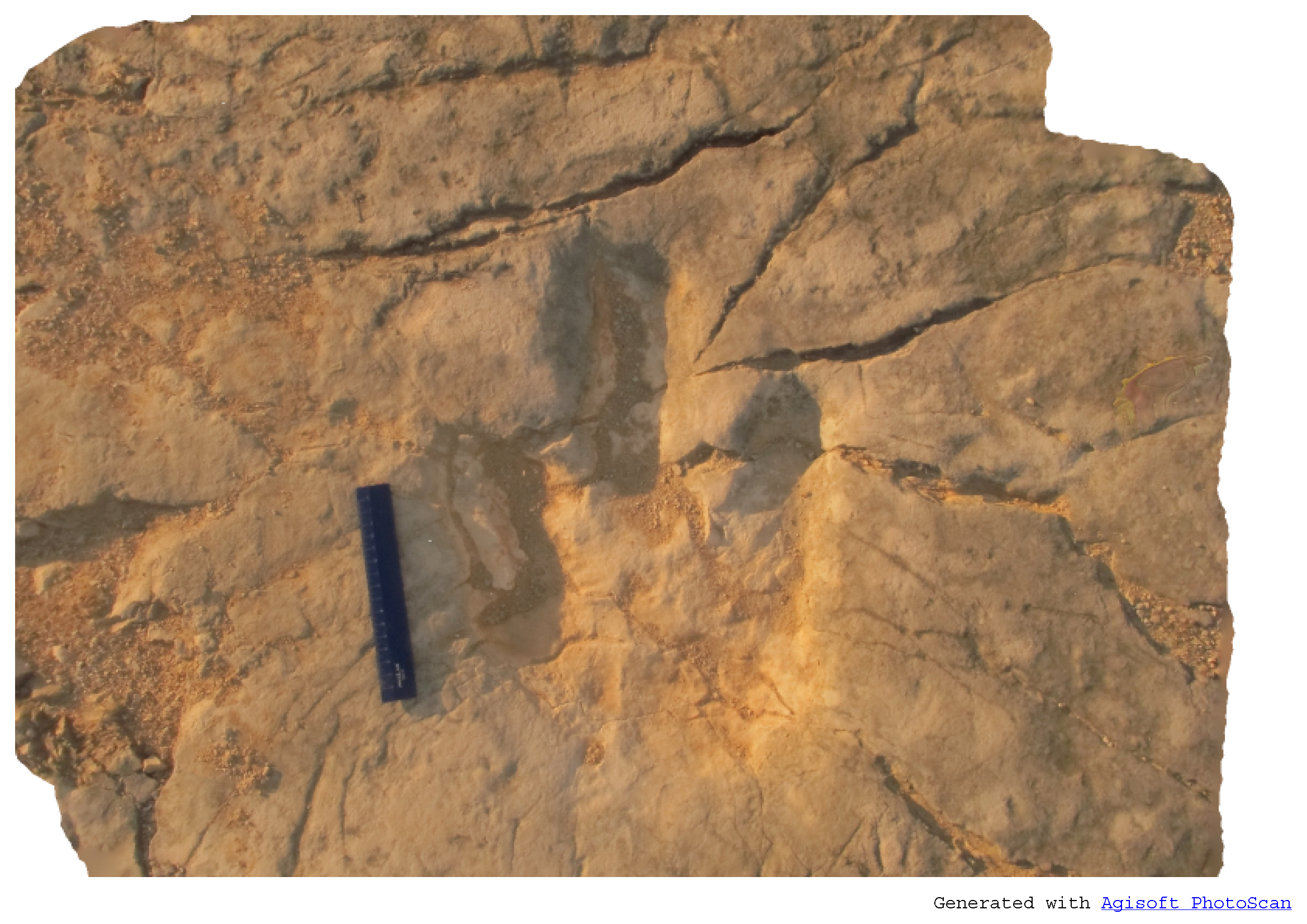


**Supplementary Data 3.** Digital reconstruction of tridactyl track VMX.1 described as *Megalosauripus* isp. from the Middle Jurassic of the Vale de Meios tracksite (Portugal) Downloadable, interactive 3D PDF file and 3D model in .ply format generated through Agisoft Photoscan software available here:

<https://figshare.com/s/37ceeda03719449a900b>

10.6084/m9.figshare.3398530


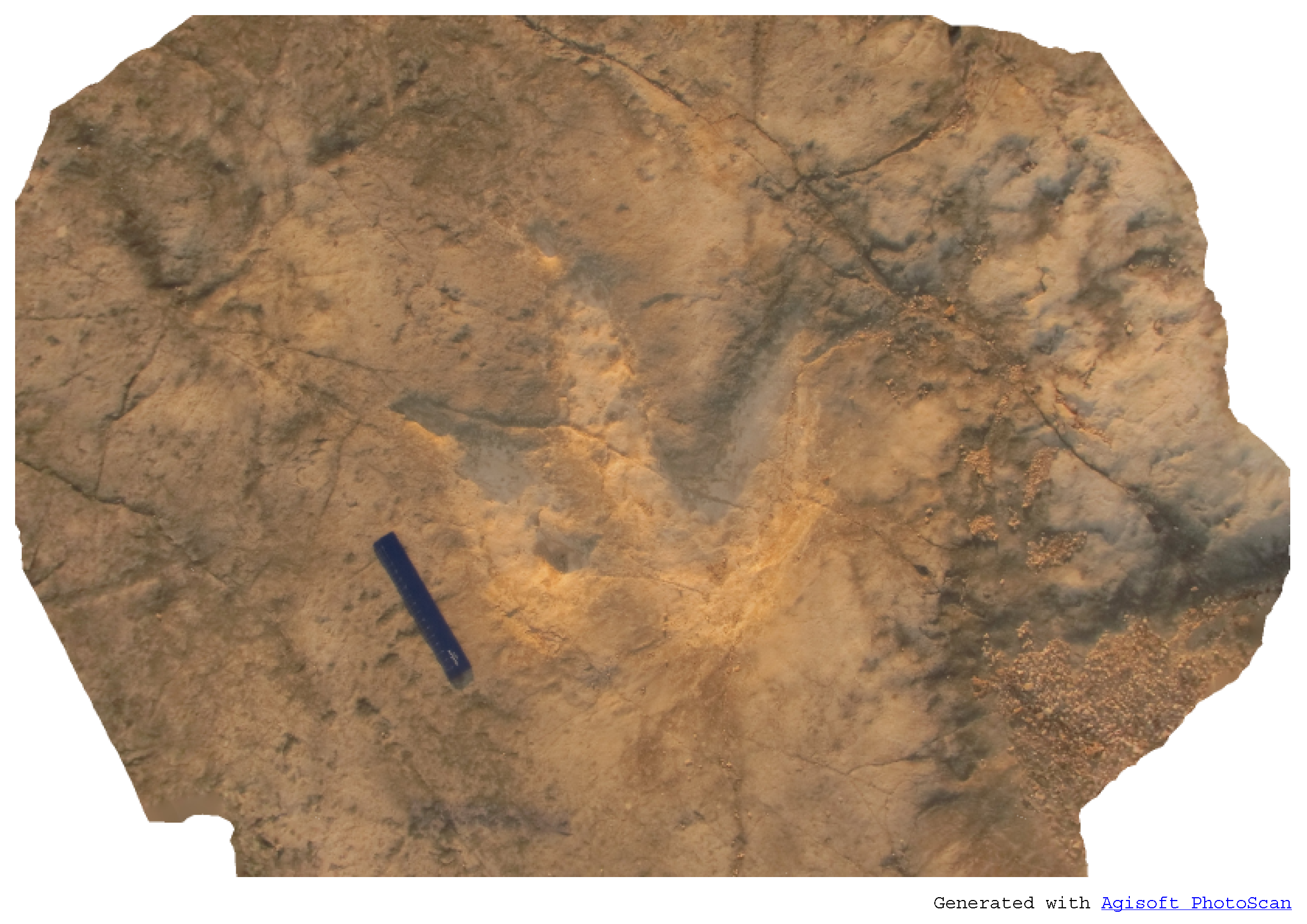


**Supplementary Data 4.** Digital reconstruction of tridactyl track VMX.2 described as *Megalosauripus* isp. from the Middle Jurassic of the Vale de Meios tracksite (Portugal) Downloadable, interactive 3D PDF file and 3D model in .ply format generated through Agisoft Photoscan software available here:

<https://figshare.com/s/33012aa15335884c3313>

10.6084/m9.figshare.3398533
